# Effectiveness of Interventions to Improve Malnutrition Among Older Adults Living with Frailty Who Are Discharged from the Acute Setting: A Systematic Review

**DOI:** 10.3390/nu17193181

**Published:** 2025-10-09

**Authors:** Cerenay Sarier, Siobhan Walsh, Sheila Bowers, Margaret O’Connor, Ahmed Mohamed, Heather Keller, Katherine L. Ford, Rose Galvin, Anne Griffin

**Affiliations:** 1 School of Allied Health, Faculty of Education and Health Sciences, University of Limerick, V94 T9PX Limerick, Ireland; rose.galvin@ul.ie (R.G.); anne.griffin@ul.ie (A.G.); 2 Nutrition and Dietetics Department, University Limerick Hospital, V94 F858 Limerick, Ireland; siobhan.walsh12@hse.ie (S.W.); sheila.bowers@hse.ie (S.B.); 3 Ageing Research Centre, Health Research Institute, University of Limerick, V94 T9PX Limerick, Ireland; oconmargaret@gmail.com (M.O.); abmohame@tcd.ie (A.M.); 4 University Limerick Hospital Group, Department of Ageing and Therapeutics, V94 F858 Limerick, Ireland; 5 School of Medicine, University of Limerick, V94 T9PX Limerick, Ireland; 6 Department of Kinesiology and Health Sciences, University of Waterloo, Waterloo, ON N2L 3G1, Canada; heather.keller@uwaterloo.ca (H.K.); katherine.ford@uwaterloo.ca (K.L.F.)

**Keywords:** older adults, nutrition intervention, care transition, malnutrition, frailty

## Abstract

Background & Aim: Malnutrition and frailty are prevalent among older adults following discharge from acute care, including emergency departments. This transition period presents a critical window for targeted nutrition interventions. This systematic review synthesises evidence on the effectiveness of nutrition interventions for malnourished, frail older adults and incorporates analyses of stakeholders’ perspectives, including those of patients, caregivers, and healthcare professionals. By integrating clinical outcomes with stakeholder experiences, the review aims to identify strategies that can optimise nutritional care and support recovery in the post-acute setting. Methods: Searches were conducted in Scopus, CINAHL, EBSCO, EMBASE, and PubMed for randomised controlled trials (RCTs) of nutrition interventions in participants ≥65 years living with frailty and identified as malnourished on discharge from acute care. The primary outcome was assessing the effects of nutrition interventions on malnutrition, nutrition status, physical function and frailty, food intake, and quality of life. Secondary outcomes were hospital readmission and mortality. The quality of studies was assessed using the Cochrane Risk of Bias Tool (V2). Results: Five RCTs with 551 participants were included. Nutrition interventions, including counselling, oral nutrition supplements, and multidisciplinary strategies, improved dietary intake, weight, frailty, physical function, BMI, and quality of life in older adults post-discharge. Some studies also reported reduced hospital stays, readmissions, and mortality. However, none explored stakeholder perspectives, highlighting a gap in person-centred transitional care design. Conclusion: This systematic review highlights a critical gap in evidence for nutrition interventions targeting frail older adults at hospital discharge. While short-term benefits were observed, long-term sustainability and real-world feasibility remain uncertain. The absence of stakeholder involvement further limits person-centred design. These findings underscore the need for integrated nutrition care pathways that embed effective interventions into transitional care models.

## 1. Introduction

Malnutrition and frailty are interrelated conditions commonly observed in older adults, both associated with increased risk of hospital readmission, functional decline, and reduced quality of life [1,2,3,4,5,6]. Malnutrition, as defined by the European Society for Clinical Nutrition and Metabolism (ESPEN), is a state of insufficient intake or uptake of nutrition leading to altered body composition, impaired function, and adverse outcomes [1,3]. Malnutrition is an umbrella term that encompasses both undernutrition and overnutrition [1]; however, in this study the focus is on undernutrition, given its close association with frailty and adverse outcomes in older adults [1,3]. Diagnosis requires initial screening for nutritional risk, followed by confirmation through measures such as low body mass index (BMI < 18.5 kg/m^2^), unintentional weight loss (>10% within 6 months or >5% within 3 months), or reduced fat-free mass [3]. Frailty is a clinically recognisable state of vulnerability arising from age-related declines across multiple physiological systems, reducing resilience to stressors. Fried et al. operationalised frailty using five criteria, grip strength, fatigue, walking speed, activity level, and weight loss, with the presence of three or more indicating frailty [4].

Frailty and malnutrition frequently coexist, sharing features such as muscle loss and functional decline that increase vulnerability during care transitions [5]. Optimal nutrition is essential not only for preventing disease but also for supporting independence throughout life [6]. Recently, studies investigating the relationship between nutritional status and frailty in older adults have indicated that nutritional risk is highly associated with frailty. In fact, inadequate nutrient intake increases dependency among older individuals and the need for care, resulting in fatigue, reduced quality of life, and increased hospitalisation rates [5,6,7]. Depending on the assessment method used, the prevalence of malnutrition in hospitalised patients ranges from 20% to 50%, but it often remains under recognised and insufficiently addressed during inpatient care [8]. According to the SHARE study, frailty prevalence among older adults varies considerably, ranging from 6% to 44% across European countries [9].

In Ireland, administrative data show high prevalence rates of frailty (42%) and moderate functional dependency (48%) among community-dwelling older adults receiving home support [10,11]. Another Irish study found that 28% of older adults admitted to emergency departments with low-urgency, medically stable, and cognitively intact presentations were at risk of malnutrition [12].

Despite their prevalence, malnutrition and frailty are often overlooked during hospital-to-home transitions, where shorter lengths of stay further limit opportunities for in-hospital interventions [13,14]. ESPEN guidelines recommend prevention and management through dietary counselling, tailored interventions, and mitigation of modifiable risk factors [15]. Evidence supports strategies such as oral nutritional supplements (ONS), dietary advice, food fortification, and mealtime interventions, with ONS shown to improve nutritional status and, when combined with dietary counselling, reduce mortality [16,17,18,19,20,21]. Dietitian-led interventions post-discharge also increase dietary intake, body weight, and nutritional status [22,23,24,25,26].

Transitional care programmes aim to improve continuity between hospital and community services, but nutrition is rarely a central focus despite its importance for physical outcomes [27]. A theory-driven evaluation approach, incorporating the perspectives of healthcare providers and older adults, can clarify how specific components such as nutritional support, exercise, and social engagement influence outcomes [28,29]. While research has examined nutrition interventions in older adults, few studies have targeted those living with frailty after hospitalisation, a group facing unique challenges including heightened vulnerability and reduced daily functioning [30,31,32,33,34].

This systematic review synthesises evidence on nutritional interventions for older adults with frailty transitioning from hospital to home. It describes interventions currently implemented, identifies providers involved in delivery, incorporates stakeholder perspectives, examines intervention frequency and fidelity, and evaluates their impact on patient outcomes. By mapping current practice and gaps, this review aims to inform the development of evidence-based, patient-oriented nutritional care strategies for this vulnerable population.

## 2. Methods

### 2.1. Protocol and Registration

This systematic review followed the Cochrane Handbook for Systematic Reviews of Interventions (Version 6.3, The Cochrane Collaboration, London, UK) [35] and is reported according to the PRISMA (Version 2020, University of Oxford, Oxford, UK) checklist [36]. The protocol for this systematic review, including the research question, search strategy, inclusion/exclusion criteria, and outcomes of interest, were specified a priori and registered at PROSPERO (Registration No. CRD42024568143, National Institute for Health Research, York, UK).

### 2.2. Eligibility Criteria

Frailty was defined according to validated operational criteria (e.g., Fried phenotype [4], Clinical Frailty Scale (CFS) [37], Short Physical Performance Battery (SPPB) [38]). Malnutrition was defined as insufficient intake or uptake of nutrition resulting in altered body composition and function, assessed using validated screening tools (e.g., Short-Form Mini Nutritional Assessment (MNA-SF) [39], Malnutrition Screening Tool (MST) [40], Full-Form Mini Nutritional Assessment (MNA-FF) [41], and Icelandic Nutrition Screening Tool (ISNST) [42]). Eligibility criteria are summarised in Table 1**.**

### 2.3. Search Strategy and Data Extraction

The search was conducted in Scopus, CINAHL, EBSCO, EMBASE, and PubMed. All retrieved citations were imported to EndNote (Version 20, Clarivate, Philadelphia, PA, USA) and thereafter imported to Covidence with full references included. Covidence software (Version 2.0, Veritas Health Innovation, Melbourne, VIC, Australia) was used for evidence selection and to remove duplicate articles. If multiple publications were identified from the same study, all relevant articles were retrieved and reviewed to ensure comprehensive data extraction. Where overlapping data were presented across reports, the most complete and up-to-date version was prioritised. Selection of studies began with independent screening study titles and abstracts by one reviewer (CS) using the pre-specified inclusion and exclusion criteria. Studies were screened and checked by the other reviewers (AG, RG) based on the inclusion criteria. Conflicts were discussed among reviewers to reach consensus. Full text articles were screened by one reviewer (CS) with reference to the inclusion criteria. One reviewer (CS) contacted authors to request access to their full-text articles to avoid missing data. Data extraction included specific details about participants, intervention, concept, context, outcome, article type, country, and study methods as relevant. The protocol data extraction form was modified and revised as necessary during the data extraction process. As part of this process one reviewer independently charted the data from the retrieved articles (CS). The other reviewers (AG, RG) checked the extracted data. Any disagreements that arose between the reviewers were resolved through discussion. Data charting was conducted using Microsoft Excel Version 2011. Data were grouped by outcome and the mean difference post-intervention was reported within the intervention (pre- and post-intervention) or between-groups (intervention vs. control), with the level of significance reported and considered statistically significant if reported *p*-value was < 0.05. Where possible, baseline participant characteristics such as risk of malnutrition and current nutrition intervention was assessed in the context of relevant clinical guidelines. To evaluate intervention effectiveness in older adults with frailty, the studies assessed outcomes including malnutrition and nutritional status, physical function, food intake, and quality of life, while also incorporating stakeholder perspectives on the design, acceptability, and feasibility of the interventions.

### 2.4. Quality Assessment

The quality of methodology of included studies was assessed independently by three reviewers (CS, AG, RG), with articles reviewed in duplicate, using the Cochrane Risk-of-Bias Tool 2 (RoB 2). The RoB 2 [43] involves assessing six domains of bias for RCTs that include, sequence generation, allocation concealment, blinding, incomplete outcome data, selective outcome reporting, and other potential sources of bias. A rating of low, high, or unclear risk was assigned to each domain from each assessment. Any disagreements between reviewers were resolved through discussion, and if consensus could not be reached, a third reviewer was consulted to make the final decision.

## 3. Results

**Figure 1:**
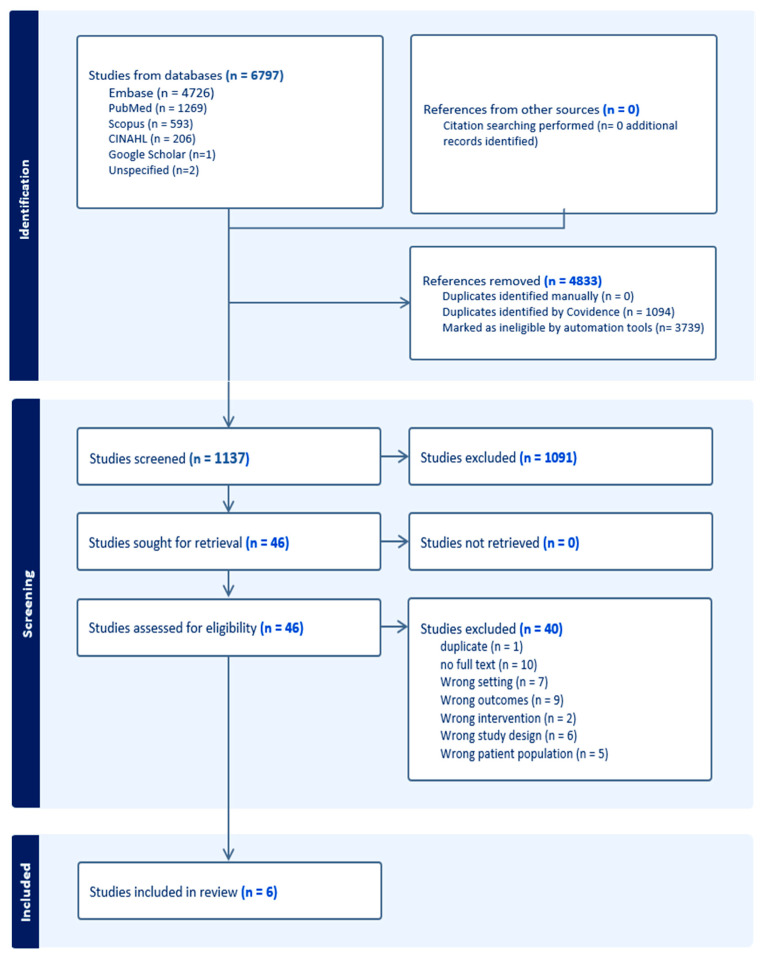
Prisma Chart.

In total, 6797 articles were identified from the databases. After removing 1094 duplicate articles and 3739 ineligible articles, a total of 1137 articles were screened based on title and abstract relevance (Figure 1). One author was contacted by email to request the full-text article, which was subsequently provided. Following full-text review, five RCTs (reported across six articles) met the eligibility criteria and were included in the review, reflecting the limited body of research in this area [33,44,45,46,47,48] (Figure 1).

### 3.1. Study Characteristics

In total, 6 articles reporting on 5 RCTs conducted between 2015 [33] and 2024 [47] were included in this review. One of the RCTs was reported across two publications [44,45]. The included RCTs had sample sizes ranging from 24 [33] to 254 [48], representing a combined total of 551 participants (Table 2). The trials were conducted in Iceland [44,45], Taiwan [46,48], Australia [33], and Denmark [47]. Two studies reported the mean age for the overall participant population (74 years [46]; 79 ± 7.7 years [33]), while three reported mean age separately for intervention (IG) and control groups (CG): IG 79.2 ± 7.0 vs. CG 80.2 ± 7.1 years [47]; IG 83.3 ± 6.7 vs. CG 81.8 ± 6.0 years [44]; IG 87.6 ± 6.0 vs. CG 85.2 ± 6.0 years [48]. Reported comorbidities included hypertension [46], diabetes [46], abnormal biochemical or medical/clinical findings [47], and dementia [48]. The healthcare settings varied across studies, including hospitals [33,44,46] and emergency departments [47,48]. Intervention duration ranged from 2 weeks [48] to 18 months [44]. The quality assessment was assessed using the Cochrane RoB 2 tool [43]. Two reviewers (CS and AG) independently assessed the risk of bias for included studies, with inconsistencies addressed through discussion. Studies were then classified as poor, fair, or good quality based on their total scores. Six of the studies were deemed overall to have a low risk of bias. The characteristics of included studies are summarised in Table 2.

### 3.2. Frailty

Frailty was assessed using varied methods across the included studies. One study relied on clinical history, focusing on functional decline and comorbidities [33], while another used the IPAQ-SF to assess physical activity as a proxy for frailty [46]. Fried’s frailty phenotype was applied in one study, incorporating measures such as weight loss, exhaustion, grip strength, walking speed, and activity levels [47]. The Clinical Frailty Scale (CFS), a judgement-based tool assessing fitness and dependence, was used in another study [48], which found significantly lower frailty prevalence in the intervention group (14.2%) compared to controls (32.6%, *p* = 0.009). Additionally, one study categorised adults with frailty into two groups: those with mechanical falls and extensive medical histories, and those with active lifestyles experiencing falls due to fainting or exertion [33]. Nutritional status varied between these groups, with most active adults with frailty found to be well-nourished, while adults with frailty who had mechanical falls more likely to be malnourished. Baseline nutritional and frailty characteristics of participants in the included RCTs are summarised in Table 3 and Table 4.

### 3.3. Characteristics of the Dietary Intervention and Control Groups

Most interventions aimed to improve nutrition status [33,46,47], dietary intake [33,46,47], malnutrition [33,45,46,47] and frailty [33,45,48]. A smaller number of studies evaluated the impact of interventions on hospital readmissions [33,44], quality of life [33,47], and mortality [44,48] after discharge. To illustrate the similarities and differences across intervention and control groups more clearly, the characteristics of dietary interventions, delivery approaches, control conditions, and follow-up care are summarised in Table 5.

### 3.4. Nutrition Status

Four studies reported the effect of the nutrition intervention on dietary intake or nutrition status [33,44,46,47]. One study showed significant weight gain in the intervention group (1.7 kg ± 2.5 kg; which equalled approximately 2% of body weight) and weight loss in the control group (−3.5 ± 3.9 kg; approximately 5% of their body weight, 42 out of 53 individuals lost >1 kg body weight) [44]. In another study, the intervention group showed an average weight gain of 0.8 ± 3.7 kg compared with weight loss of −1.1 ± 4.6 kg in the standard care group [33]. Another study showed that while there was no significant difference in baseline energy intake between the control and intervention groups (*p* = 0.412), by the study endpoint, the intervention group showed a significantly higher energy intake (2412 ± 403 kcal) compared to the control group (731 ± 320 kcal; *p* < 0.001) [44]. The intervention was successful in increasing the participants’ energy and protein intake, improving their physical and cognitive function, and their depressive symptoms in comparison to the control group [44,45].

### 3.5. Dietary Intake

Wu et al. (2018) reported that food intake of vegetables, dairy, and nuts increased in addition to increasing the concentration of urinary urea nitrogen in older adults at prefrail or frail stages [46]. The significant increase in the concentration of urinary urea nitrogen reflected an increased consumption of total protein from protein-rich foods such as beans, fish, meats, eggs, and milk. Overall, the improvement of multiple dietary components included the increased consumption of vegetables, dairy, and nuts and an adequate amount of fruit intake, along 8 weeks after discharge, the intervention group had significantly higher mean intakes of energy (30 kcal/kg/day vs. 24 kcal/kg/day, *p* = 0.012) and protein (1.2 g/kg/day vs. 0.9 g/kg/day, *p* = 0.0025) compared to the control group [46].

### 3.6. Impact of Nutrition Intervention on Frailty

Vivanti et al. (2015) compared participants who received the intervention (the standard care and also undertook individualised dietary counselling at baseline in which nutrition goals and strategies were made in collaboration with the ED dietitian following standard medical nutrition therapy practice) to those in the control group and found that 12 (19%) participants in the control group were lost to follow-up 16 weeks after discharge, compared to 20 (31%) in the intervention group. Among those who were lost to follow-up, frailty (70% vs. 55%, *p* = 0.005) and physical inactivity (71% vs. 48%, *p* = 0.02) were more prevalent compared to those who completed the 16-week follow-up. Despite these differences, baseline characteristics of those who dropped out were similar to those who completed the study [33].

Wu et al. (2018) reported that physical function changes were assessed using the Fried frailty criteria [4], revealing improvements in several frailty-related domains [46]. Gait speed improvement was most notable in Group 3 (multinutrient + soy protein; +1.12 sec/10 m) and Group 2 (multinutrient; +1.04 sec/10 m), while grip strength, particularly in the left hand, increased in all intervention groups except the control. Physical activity levels, measured by the International Physical Activity Questionnaire-Short Form (IPAQ-SF), showed the largest increase in Group 3 (+1853 kcal/week), indicating improved functional capacity and potentially reduced frailty. Notably, Group 4 (receiving nutrition education, customised dishware, and food supplements) demonstrated the greatest reduction in overall frailty score (−0.7 *p* < 0.05) at both the 1-month and 3-month follow-up points, suggesting the strongest improvement. The group receiving multinutrient also experienced a notable decrease (around −0.4), followed by the multinutrient and soy protein group with a modest decrease (approximately −0.2). The control group showed the smallest change (approximately −0.2) [46]. Also, participants who received supplements were asked to complete a self-reported daily log. The compliance rate of Group 2, Group 3, and Group 4 was 97.7%, 86.3% and 92.5%, respectively; these rates were calculated based on the log sheet [46].

### 3.7. Quality of Life and Readmissions

Two studies assessed quality of life [33,47]. In one study, the intervention group showed improvements in both quality of life and depression compared to the control group. The mean score on the EDQ5 improved by 14.4 ± 29 points in the intervention group, while it remained stable in the standard care group (−0.1 ± 16.4) [33]. In contrast, the other study found that a multidisciplinary and transitional nutritional intervention did not improve health-related quality of life, compared to standard care [47]. One study showed that the intervention group had significantly fewer readmissions (significant at 1, 6 and 12 months) and shorter length of stay (significant at all time points) when compared to the control group; however, the differences in emergency room visits were not significant [44]. Another study found that patients who received the nutrition intervention, which began during their hospital stay and continued with follow-up care, had a shorter length of hospital stay compared to those who received standard care. In addition, the intervention group experienced fewer readmissions after discharge. Outcome measures, including body weight, quality of life, depression, falls history, and days of hospital admissions, were collected at baseline and 12 weeks. While the intervention led to clinically important improvements in these outcomes, the improvements were not significant. These findings suggest that incorporating malnutrition screening in the emergency department and providing dietetic follow-up for patients at nutritional risk could be beneficial in improving patient outcomes after hospitalisation [33].

Another study observed that the participants who received the intervention (nutrition consultation provided by dietitians to patients and their caregivers helping them to prepare daily meals and physical therapy focused on strengthening exercise, endurance training, balance training, chest care, and ambulation training) for more than two weeks had lower risk of emergency department (ED) visits, readmissions and mortality. The study observed that the duration of the intervention was an independent protecting factor for ED visits (OR 0.21, 95% confidence interval [CI] 0.10–0.43; *p* = 0.024), readmissions (OR 0.30, 95% CI 0.16–0.56; *p* < 0.001), and mortality (OR 0.20, 95% CI 0.04–0.87; *p* = 0.032). Compared with the controls, the intervention group had significantly lower cumulative incidences in emergency room visits (*p* = 0.031) and mortality (*p* = 0.014) within 90 days after intervention. There was no significant difference in practice between centres (in five hospitals) with regard to readmissions and mortality [48].

## 4. Discussion

### 4.1. Statement of Principal Findings

This systematic review synthesised evidence on nutritional interventions for older adults with malnutrition and frailty following hospital discharge and found that most interventions, particularly those led by dietitians, were associated with improvements in nutritional status, dietary intake, and frailty scores [33,44,45,46,47,48]. Gains in energy and protein intake were consistently reported [45,46], often accompanied by weight gain [33,45,46] and improvements in nutritional biomarkers such as urinary urea nitrogen [46]. Some studies demonstrated additional benefits including improved physical function, quality of life, and reduced hospital readmissions [33,44,47,48], although findings in these areas were not consistent across trials. These findings indicate that dietitian-led, individualised strategies can deliver meaningful clinical benefits for older adults with frailty in the post-discharge period.

Nutritional support following hospital discharge is crucial for older adults with frailty, as evidence consistently shows benefits of maintaining weight, body composition, and physical function. Research demonstrates the positive impact of dietetic-led, individualised nutritional interventions on maintaining and improving nutrition status. Dietetic expertise allows for interventions that address comorbidities, functional limitations, and patient goals, supporting recovery while maintaining dignity and autonomy [49]. Targeted, individualised approaches using ONS, high-energy and high-protein meals, and dietetic counselling have been shown to prevent post-discharge weight loss, support gains in lean mass, and improve mobility and self-reported function [33,45,46,50,51,52,53]. Broader evidence from large trials, including the EFFORT study, further demonstrates that individualised nutritional support aimed at meeting energy and protein targets improves quality of life, reduces hospital readmissions, and can positively influence mortality [54,55,56]. These results suggest that dietitian-led nutritional care during the transition from hospital to home is an essential component of recovery and independence for older adults [57].

Evidence consistently indicates that older adults with frailty are particularly vulnerable to poor nutritional intake following hospital discharge, which contributes to malnutrition and delayed recovery. Interventions that combine high-energy, high-protein foods with personalised dietary counselling, ONS, and supportive strategies have been shown to improve nutritional intake, physical function, and quality of life. Studies further highlight that individualised, dietitian-led approaches can reduce frailty, enhance mobility, and support functional recovery, reinforcing the importance of tailored nutritional strategies in this population [58,59,60,61]. Our findings align with previous reviews showing that high-energy, high-protein foods and tailored counselling can enhance intake and prevent deterioration [62]. Other studies demonstrated that individualised strategies such as personalised counselling and customised dishware improved intake and supported functional recovery [45,46,63,64]. These approaches emphasise the value of dietitian input in tailoring care to individual needs and capacities.

In a three-month intervention study, Rydwik, Frändin, and Akner found that community-dwelling older adults with frailty receiving personalised dietetic intervention demonstrated improvements in gait speed and habitual physical activity [65]. Similarly, our findings observed that participants who received individualised nutrition education experienced a greater reduction in frailty scores compared to other intervention groups [33,45,46]. Nykänen et al., observed that older adults receiving individual dietary counselling consisting of two in-person sessions with a dietitian and bi-monthly follow-up calls over one year showed improvements in frailty status and nutritional assessment scores relative to a control group [64]. Taken together, these findings suggest that dietary pattern modification, particularly when incorporating multiple nutritional strategies, may play a key role in reversing frailty.

Beyond the clinical outcomes identified in this review, it is also important to consider the broader health economics and outcomes research (HEOR) and real-world evidence (RWE) that highlight the value of nutritional interventions. HEOR studies have demonstrated that dietitian-led care and the use of ONS can reduce healthcare utilisation and costs by preventing complications and hospital readmissions [66,67,68,69,70,71]. RWE further supports the feasibility of integrating these interventions into transitional care models, showing how they can be scaled and sustained in practice [72,73]. Together, these perspectives strengthen the case for nutrition interventions as strategies that deliver both clinical benefit and economic value. Overall, this review reinforces the critical role of individualised nutritional interventions in supporting recovery and well-being among this vulnerable population.

Although several studies used tools and approaches consistent with person-centred care, none explicitly reported stakeholder perspectives, such as patient or caregiver views of nutritional support during transitions. This represents an important gap in the literature, as involving stakeholders is essential for designing models of transitional nutrition care that are both effective and acceptable to older adults living with frailty.

### 4.2. Strengths and Limitations of the Review

A key strength of this review is the generally high methodological quality of the included studies, with most RCTs assessed as having a low risk of bias, which enhances confidence in the reliability of the reported effects. The review also provides a comprehensive synthesis of interventions delivered across a range of post-discharge settings, highlighting the role of dietitian-led, individualised nutritional strategies in supporting older adults with frailty.

However, several limitations should be acknowledged. Only five RCTs met the eligibility criteria, reflecting the limited research base in this area. This small number of included studies, combined with modest sample sizes, resulted in limited statistical power to detect differences in important outcomes such as readmissions, mortality, and quality of life. Furthermore, none of the included trials reported patient- or caregiver-reported outcomes (e.g., quality of life, satisfaction, or caregiver burden), representing an important evidence gap in understanding the full impact of post-discharge nutrition care.

The considerable heterogeneity across the included studies warrants attention. Interventions differed in duration, intensity, delivery methods, and the professional backgrounds of those providing nutrition care, while outcome measures varied substantially across trials. This variability prevented the conduct of a meta-analysis and reduced comparability between studies, limiting the ability to draw firm conclusions about which components of nutritional care are most effective. Such heterogeneity also constrains the generalisability of the findings, making it difficult to translate results into standardised models of practice for older adults with frailty following hospital discharge.

While RCTs are the gold standard for establishing effectiveness, more robust, adequately powered trials with longer follow-up are needed to establish the sustainability of benefits and to identify the most effective components of nutritional care [33,44,45,46,47,48]. Future research should also incorporate standardised intervention protocols, core outcome measures, and patient- and caregiver-reported outcomes to strengthen the evidence base and ensure that evaluations capture outcomes that matter most to those receiving care [74]. Despite these limitations, this review reinforces the critical importance of individualised nutritional interventions in promoting recovery, functional outcomes, and well-being among older adults with frailty.

### 4.3. Implications of Results for Practice, Policy, and Future Research

This review highlights the critical role of dietitian-led, individualised nutritional care in supporting older adults with frailty following hospital discharge. By tailoring interventions to individual needs, dietitians can help maintain nutritional status, support functional recovery, and enhance quality of life. Integrating nutrition care into discharge planning, including routine screening, assessment, and personalised care pathways is essential to address the unique vulnerabilities of this population.

From a policy perspective, findings support the development of structured nutrition pathways across acute and community settings. Allocating resources for ongoing dietetic follow-up, nutritional supplements, and home-delivered meals can help ensure continuity of care and reduce post-discharge complications. Quality improvement frameworks, such as FOCUS (F = Find a problem, O = Organise a team, C = Clarify the problem, U = Understand a problem, S = Select an intervention) and PDSA (P = Plan, D = Do, S = Study, A = Act) (FOCUS-PDSA) [75,76,77,78], may facilitate the embedding of nutrition interventions into routine practice, enabling iterative testing, refinement, and sustainable implementation.

Future research should focus on the long-term effectiveness, cost-effectiveness, and scalability of dietitian-led interventions. Importantly, capturing the perspectives of key stakeholders including patients, caregivers, and healthcare professionals is essential to design feasible, acceptable, and context-sensitive care models. Greater attention to the transitional period from hospital to home is warranted, as this remains a high-risk for malnutrition and adverse outcomes among older adults with frailty.

## 5. Conclusions

This review identifies a clear gap in research on nutrition interventions for older adults with frailty at hospital discharge, limiting the development of evidence-based care plans. Dietitian-led, individualised nutritional strategies combining high-protein, high-energy meals with tailored education and ongoing support emerge as the most promising approach, improving dietary intake, body weight, physical function, and frailty outcomes. However, the evidence remains limited by small-scale studies and heterogeneity in interventions. Future research should prioritise high-quality, long-term trials, incorporate real-world implementation and stakeholder perspectives, and evaluate both clinical and economic outcomes. Embedding nutrition interventions within structured quality improvement frameworks may support sustainability, scalability, and integration into transitional care pathways.

## Figures and Tables

**Table 1:** Inclusion criteria according to PICO (population, intervention, comparator and outcome).

Domain	Inclusion Criteria	Exclusion Criteria
Population	Adults aged ≥ 65 years living with frailty and identified as malnourished at discharge from acute care settings	Individuals < 65 years or without frailty and malnutrition
Intervention	Nutrition-based interventions (e.g., ONS, dietary counselling, meal programmes)	Interventions without a nutrition component
Comparator	Standard care or other active interventions	No comparator group
Outcomes	Primary; change in nutritional status. Secondary; physical function, frailty score, food intake, quality of life, hospital readmission, mortality and stakeholders’ perspectives on the model of nutritional care (e.g., acceptability, feasibility, perceived benefits and challenges, experiences, and priorities of patients, caregivers, clinicians, and decision-makers).	No relevant outcomes reported
Setting	Discharged from acute care settings (e.g., hospital wards, emergency departments)	Long-term care residents, nursing home patients, or those not discharged from acute care
Study Design	Randomised controlled trials (RCTs) or studies with a randomised design	Non-randomised studies, observational studies, case reports
Language	Any language with a full English translation or full English text	Studies without English abstract or inaccessible full text

**Table 2:** Characteristics of Studies Included in the Systematic Review.

Author	Participants	Sex	Mean Age	Healthcare Setting	Comorbidities
[44]	N = 106 > 65 years IG: *n* = 53 CG: *n* = 53	IG: 71.7% F CG: 52.8% F	IG: 83.3 ± 6.7 CG: 81.8 ± 6.0	Discharge home from the hospital within a day of recruitment	N/A
[45]	N = 106 > 65 years IG: *n* = 53 CG: *n* = 53	IG: 71.7% F CG: 52.8% F	IG: 83.3 ± 6.7 CG: 81.8 ± 6.0	Patients were discharged home to independent living from the hospital	N/A
[46]	N = 37 pre-frail or frail Modified L. Fried criteria; without severe disease (e.g., cancers under treatment, immobilisation, or severe arthritis), diagnosed dementia, mental illness, or an inability to communicate	N = 17 M N = 20 F	74	Hospital	Hypertension Control: 6 (60%) Group 2: 6 (75%) Group 3: 5 (55.6%) Group 4: 6 (66.7%) Diabetes Control: 2 (20%) Group 2: 3 (37.5%) Group 3: 2 (22.2%) Group 4: 3 (33.3%)
[33]	N = 24 >60 years screening positive for risk of malnutrition using the malnutrition screening tool excluding category 1, nursing home resident or under dietetics already	42% M	79.0 ± 7.7	Emergency Department	N/A
[47]	N = 130 IG *n* = 65 CG *n* = 65	CG: n (%): 26(36.9) n (%): 26(36.9) IG:	CG: 80.2 (7.1) IG: 79.2 (7.0)	Emergency Department	CG: Abnormal clinical and laboratory finding n(%): 8 (13.1), Diseases of respiratory system: 23 (37.7), Other diseases 30 (49.2) IG: Abnormal clinical and laboratory finding n(%): 12 (21.8), Diseases of respiratory system: 18 (32.7), Other diseases 25 (45.5)
[48]	N = 254	IG M: 101 (49.3%) CG M:28 (57.1%)	IG: 87.6 ± 6.0 CG: 85.2 ± 6.0	Emergency Department	The most prevalent diagnosis was dementia (40.5%) for the PAC group and CKD (73.5%) for the control group (*p* < 0.001)

N/A: Not applicable; indicates that the information was not relevant or not reported for the corresponding study/variable.

**Table 3:** Baseline Nutritional Status of Participants in Included RCTs.

Study	Nutritional Measure	Control (CG)	Intervention (IG)
[44,45]	ISNST score	4.5 ± 1.3	5.1 ± 1.7
	BMI (kg/m^2^)	26.9 ± 5.3	28.5 ± 6.5
	Fat-free mass (kg)	49.1 ± 11.9	48.1 ± 10.2
[46]	BMI (kg/m^2^)	24.6 ± 1.1	25.5 ± 0.9 (multinutrient)/25.5 ± 1.1 (multinutrient + soy)/28.4 ± 1.2 (nutrition education)
	MNA-SF (% normal)	100%	100%/88.9%/100%
[33]	MST ≥ 2	–	88%
[47]	Body weight (kg)	70.9 ± 19.4	69.5 ± 15.9
[48]	MNA (% normal)	29.8%	8.9%

CG: Control Group; IG: Intervention Group; MST: Malnutrition Screening Tool; MNA: Mini Nutritional Assessment; ISNST: Icelandic Nutrition Screening Tool. ISNST: (cut-off ≥ 4 = at risk of malnutrition). BMI: Body Mass Index (kg/m^2^)—underweight < 18.5; normal 18.5–24.9; overweight ≥ 25; obese ≥ 30. MNA-SF: Mini Nutritional Assessment–Short Form (12–14 = normal; 8–11 = at risk; 0–7 = malnourished). MST: (≥2 = at risk of malnutrition). MNA (full form): 24–30 = well nourished; 17–23.5 = at risk; <17 = malnourished.

**Table 4:** Baseline Frailty/Functional Status of Participants in Included RCTs.

Study	Frailty/Functional Measure	Control (CG)	Intervention (IG)
[44,45]	Handgrip strength (kg)	21.5 ± 8.5	19.7 ± 6.8
	SPPB score	2.4 ± 2.0	2.5 ± 1.8
	Pre-frail (%)	80	87.5/77.8/88.9
[46]	Frail (Fried’s criteria)	62.5%	71.9%
[33]	Frailty (CFS) Mild Moderate Severe	32.6% 28.6%38.8%	14.2% 35.1%50.7%
[47]	ADL dependence IADL dependence	5 (10.2%) 0 (0%)	28 (13.7%) 7 (3.4%)

SPPB: Short Physical Performance Battery; CFS: Clinical Frailty Scale; ADL: Activities of Daily Living; IADL: Instrumental ADL. SPPB: (0–12 points; ≤9 = mobility limitation/frailty risk). Fried’s criteria: Frailty phenotype defined by ≥3 of 5 (weight loss, exhaustion, weakness, slowness, low activity). CFS: (1–3 = very fit to managing well; 4 = vulnerable; 5 = mildly frail; 6 = moderately frail; 7+ = severe frailty). ADL: (range 0–5; lower scores indicate more dependent). IADL: (range 0–8; lower scores suggest more dependent).

**Table 5:** Characteristics of Dietary Approaches and Control Groups in Included Studies.

Study (Ref.)	Intervention Team	Screening Tools	Physical Function Measures	Intervention Characteristics	Control Group	Follow-Up Care and Duration	Primary Outcome(s)	Secondary Outcome(s)
[44]	Clinical nutritionists	MNA-FF, ISNST	SPPB	Individual therapy: 5 in-person + 3 phone calls; home-delivered energy/protein-rich meals (1 hot meal + 2 snacks/day, with/without ONS)	Nutrition recommendations for older adults; encouraged Meals on Wheels (MOW)	Community follow-up; home visits; 1-, 6-, 12-, and 18 months post-discharge	Hospital readmissions and Length of stay	Mortality and need for long-term care residence
[45]	Clinical nutritionists	ISNST	SPPB	Nutrition counselling for community-dwelling older adults	Nutrition recommendations + encouraged MOW	Community/primary care setting; 6 months	Energy- and protein intake, body weight and physical function	Anthropometric measurements, nutritional status, muscular strength, dietary intake, exercise, and reported food-related digestion issues, such as diarrhoea, nausea, constipation, or stomach pain.
[46]	Registered dietitian, trained researchers	MNA-SF, MST	Grip strength, gait speed	Four groups: (1) Daily food guide; (2) + micronutrient supplements; (3) + micronutrients + soy protein; (4) + individualised nutrition education, customised dishware, food supplements	Daily food guide	Community/primary care setting; 1 month and 3 months	Dietary intake	Comprehensive geriatric assessment including a nutritional status assessment, modified L. Fried’s frailty assessment, and depressive symptoms assessment
[33]	Registered dietitian, MDT (nurses, geriatricians, PT, OT, speech therapists, pharmacists, social workers)	Not reported	Not specified	Individualised dietary counselling	No information on standard care	Yes, post-discharge; 12 weeks	Body weight, quality of life, depression, falls history and days of hospital admissions	
[47]	Registered dietitian, MDT	MNA-SF	STS test	Individualised dietary counselling	Standard care: daily dietary records validated by dietitian	Yes, post-discharge; 8 and 16 weeks after discharge	Health-related quality of life	Intake of energy and protein, body weight, well-being, hand grip strength, frailty
[48]	Registered dietitian, MDT	Not reported	CFS	Individualised dietary counselling	Dietary counselling during hospitalisation	Yes, post-discharge; 90 days	Emergency room visits, readmissions, and mortality	

## Data Availability

No additional data, code, or materials are publicly available. Data extracted from included studies are available from the corresponding author upon reasonable request.
